# Prediction of Joint Space Narrowing Progression in Knee Osteoarthritis Patients

**DOI:** 10.3390/diagnostics11020285

**Published:** 2021-02-11

**Authors:** Charis Ntakolia, Christos Kokkotis, Serafeim Moustakidis, Dimitrios Tsaopoulos

**Affiliations:** 1 Department of Computer Science and Biomedical Informatics, University of Thessaly, 35131 Lamia, Greece; cntakolia@uth.gr; 2 Institute for Bio-Economy & Agri-Technology, Center for Research and Technology Hellas, 38333 Volos, Greece; c.kokkotis@certh.gr; 3 Department of Physical Education & Sport Science, University of Thessaly, 42100 Trikala, Greece; 4 AIDEAS OÜ, Narva mnt 5, 10117 Harju Maakond, Estonia; s.moustakidis@aideas.eu

**Keywords:** machine learning, knee osteoarthritis, joint space narrowing prediction, feature selection, interpretation

## Abstract

Osteoarthritis is a joint disease that commonly occurs in the knee (KOA). The continuous increase in medical data regarding KOA has triggered researchers to incorporate artificial intelligence analytics for KOA prognosis or treatment. In this study, two approaches are presented to predict the progression of knee joint space narrowing (JSN) in each knee and in both knees combined. A machine learning approach is proposed with the use of multidisciplinary data from the osteoarthritis initiative database. The proposed methodology employs: (i) A clustering process to identify groups of people with progressing and non-progressing JSN; (ii) a robust feature selection (FS) process consisting of filter, wrapper, and embedded techniques that identifies the most informative risk factors; (iii) a decision making process based on the evaluation and comparison of various classification algorithms towards the selection and development of the final predictive model for JSN; and (iv) post-hoc interpretation of the features’ impact on the best performing model. The results showed that bounding the JSN progression of both knees can result to more robust prediction models with a higher accuracy (83.3%) and with fewer risk factors (29) compared to the right knee (77.7%, 88 risk factors) and the left knee (78.3%, 164 risk factors), separately.

## 1. Introduction

Osteoarthritis is the most common form of arthritis while the knee is the most frequently affected joint [1]. Knee osteoarthritis (KOA) is a chronic disease that can lead to joint damage, pain, stiffness, and loss of physical function. These physical limitations have a negative impact to the social life, mental health, and quality of life of KOA patients [1,2]. Due to the heterogeneity of patients’ characteristics and the multifactorial nature of KOA, the pathophysiology of the disease remains poorly understood, setting the necessity for the development of diagnostic and predictive tools.

The diagnosis or even the treatment of KOA is still a challenge for the scientific community. However, the increasing amount of medical data related to KOA permitted the development of more recent studies by using artificial intelligence and big data. According to our knowledge, few studies in the literature have adopted advanced analytic techniques such as machine learning (ML) models, to predict the development of KOA [3,4].

In 2018, Du et al. employed four ML techniques to predict the progression of KOA on MRI by using the change of Kellgren and Lawrence (KL) grade, joint space narrowing on medial compartment (JSM) grade, and joint space narrowing on lateral compartment (JSL) grade as progression metrics [5]. Lazzarini et al. [6] focused on the identification of key variables (including biomarkers) and their incorporation within predictive models of KOA. They used five outcome measures of incident KOA, including medial joint space narrowing (JSN). The study was limited to overweight and obese women. In [7], self-reported knee pain and radiographic assessments of joint space narrowing were used to cluster the OA progression and develop models for early KOA prediction. Similarly, Tiulpin et al. presented a multi-modal ML-based KOA progression prediction model using clinical examination results, raw radiographic data, and the medical history of patients. They demonstrated an improved subject selection process [8]. Furthermore, Nelson et al. applied innovative ML approaches to KOA phenotyping [9]. They used data from the FNIH Biomarkers Consortium and identified key variables that are associated with a progression phenotype. In [10], the authors used MRI images and biomechanical data in order to develop a multidimensional platform for KOA prediction. This work contributed to the improvement of OA outcome prediction and stratification of patients. This study constitutes the first attempt to a large-scale integration of skeletal biomechanics and compositional imaging.

In [11], Kellgren and Lawrence grading schemes were adopted to compare the performance of a statistical model based on patient’s questionnaire data and a ML model based on X-ray images, with respect to their prediction accuracy. The results showed that a combination of both approaches could lead to better performance. Widera et al. [12] studied a multi-class problem regarding the prediction of KOA progression. The authors used clinical data and X-ray image assessment metrics and investigated various algorithms and learning process configurations. In another study, Wang et al. used a long short-term memory model to predict KOA severity [13]. They presented a 90% accuracy of the KL grade prediction of the patient’s next visits and they demonstrated that JSN was a major contributor to KOA progression. Moreover, Lim et al. used statistical data in a deep neural network with scaled Principal Component Analysis (PCA) for the early detection of KOA [14]. Furthermore, Brahim et al. presented a computer aided diagnosis (CAD) system for early KOA detection employing X-ray imaging and ML algorithms [15]. The proposed method achieved an 82.98% accuracy.

Alexos et al. [16] investigated the progression of pain in KOA patients using clinical data collected only at the baseline. Specifically, they proposed a robust feature importance voting system for identifying the most important risk factors with an accuracy of up to 84.3%. Furthermore, Kokkotis et al. [17] demonstrated a ML-based methodology capable of predicting KOA progression, specifically KL grades progression. Notably, they worked on the identification of important risk factors that contribute to the prediction of KOA with a 74.07% accuracy. A machine learning pipeline was proposed in [18] for predicting the JSN progression in KOA patients for the right and left knee. The predictive models with the higher accuracy proved to be an SVM model for the right knee (77.7% accuracy) and a linear regression model for the left knee (78.3% accuracy). In addition, Jamshidi et al. proposed a ML methodology for the identification of important risk factors that are associated to KOA incidents [19]. They used data from OAI and they concluded that baseline X-ray and MRI-based features could identify early OA knee progressors.

Conventional approaches for identifying the risk factors for the prediction of KOA progression incorporate mostly tedious and time-consuming image-based methods. Therefore, there is a need for more efficient and explainable methods that could support clinical decision making and could enable the early detection of individuals who are likely to present severe KOA. The great majority of the aforementioned are based on images and are applying image-based processing (e.g., CNN (convolutional neural network) or other deep learning techniques) for prediction or diagnosis. Studies with exclusively non-imaging data and processing techniques have been only published on the KOA diagnosis task.

To the best of our knowledge, no work has been performed for the development of ML prediction models to predict the JSN on Medial compartment (JSM) progression for healthy patients via highly dimensional features coming from multiple heterogenous data sources. The development of explainable models could enable accurate decision making on medical examination via early detection of healthy individuals that are expected to develop knee osteoarthritis in the future.

Hence, this highlights the need for a further study and development of new techniques for determining risk factors that contribute to the development of accurate and reliable tools for predicting KOA. This study aims towards the accurate prediction of JSN on Medial compartment (JSM) progression via the development of a novel machine learning approach. This ML approach handles the heterogeneity among a plethora of features (725) deriving from various feature categories, including diagnosis from medical examination and medical imaging outcomes, among others. In this study the effectiveness of two strategies is investigated for predicting the JSN progression of KOA patients by: (i) Developing predictive models that are trained on data from the left knee and right knee separately and (ii) developing predictive models that combine KOA patients’ data for both the right and left knee. For each strategy the same steps were followed. Initially, a clustering approach is applied for the identification of patients groups with and without JSN progression. Then the risk factors are identified based on a voting scheme that incorporates various categories of feature selection techniques. The prediction stage was implemented with the use of well-known ML models in an extensive comparative experimentation. Post-hoc explainability was finally explored using SHapley Additive exPlanations (SHAP) to rank features in terms of their impact on the final ML outputs.

The paper is organized as follows. In Section 2, the proposed methodology along with the necessary data pre-processing, feature selection, and validation mechanisms, are presented. Section 3 gives a description of the medical dataset that was used in our paper along with the evaluation methodology that was followed. Results are given in Section 3. Conclusions and future work are finally drawn in Section 4.

## 2. Materials and Methods

### 2.1. Methodology

**Figure 1:**
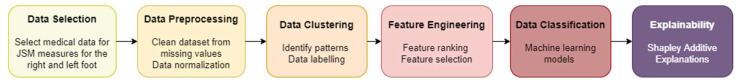
Methodology flowchart.

A machine learning approach was developed in this work by taking advantage of the combination of predictive and descriptive techniques, such as clustering, FS, and classification. The proposed methodology for predicting JSN consists of 5 main steps: (i) Data pre-processing, (ii) data clustering, (iii) feature selection, (iv) data classification, and (v) post-hoc explainability with SHAP. In the first step, data cleaning and normalization are performed to remove noise and bring all the variables to the same range. Then the samples are clustered based on their JSN progression using well-known clustering algorithms. Then, a selection of features is realized based on the identified clusters (that are considered as classes in our case). The selected features are used to develop prediction models for the KOA progression of patients (Figure 1).

In this study two strategies were investigated: (i) In the first one, two predictive models were developed using data from the right and the left knee, separately and (ii) the second strategy focuses on the development of a unique predictive model using data from both knees of KOA patients.

#### 2.1.1. Data Pre-Processing

Data deletion was performed by excluding the columns with more than 20% missing values compared to the total numbers of subjects. Furthermore, data imputation was implemented to replace missing values of categorical or numerical variables by the most frequent value of the non-missing variables. In addition, a common requirement for many ML classifiers is the standardization of the dataset. In this work, data were normalized to [0, 1] to build a common basis for the FS algorithms that follow.

#### 2.1.2. Data Clustering

**Figure 2:**
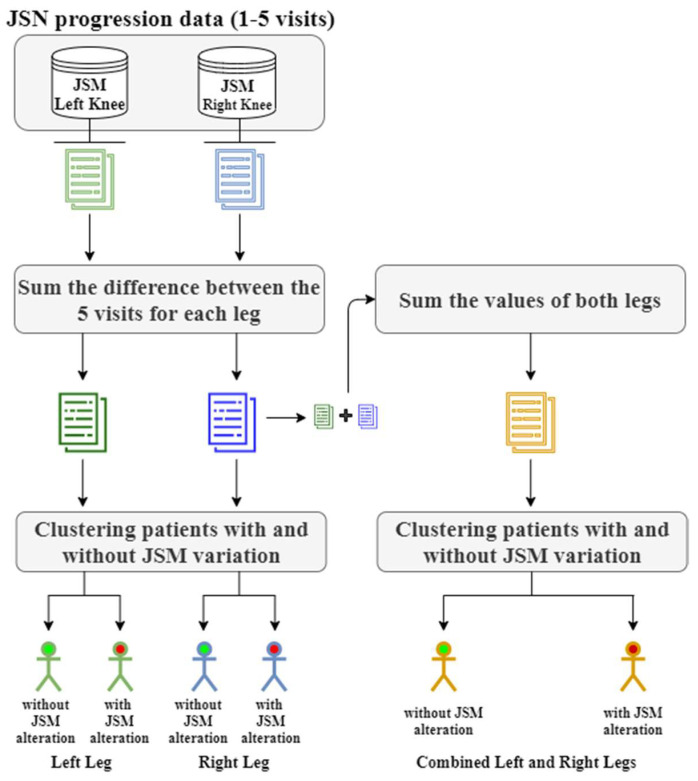
Clustering process of the proposed methodology. JSM: Joint space narrowing on medial compartment.

In this study, the clustering process that was followed is presented in Figure 2 and is described by the pseudocode in Algorithm 1. As a first step, for each patient *p* ∈ 𝒫, where 𝒫 is the set of the patients included in the cleaned medical data, the differences between the consecutive measurements are calculated:

(1) $$d_{j}^{p}=m_{j}^{p}-m_{i}^{p},\;\forall i\in \left[1,\ldots ,n-1\right],\;j\in \left[2,\ldots ,n\right]$$

where $m_{i}$ is the ith JSN measurement and n is the number of the measurements performed for the examined knee (left or right). In the presented case study, 5 measurements were used for each leg that show the JSN progression through the first 5 visits. The absolute sum of the differences is then calculated:

(2) $$\overset{n}{\underset{k=2}{\sum }}\left|d_{k}^{p}\right|$$

forming an indicator of the JSN progression within the first 5 visits for each knee. These values were used for performing the clustering.

In case of the second clustering strategy, we denote with *p_l_* ∈ 𝒫, the medical data from patient p for the left (l) knee, and with *p_r_* ∈ 𝒫 the medical data from patient p for the right (r) knee. The sum of the absolute sum of each leg was calculated:

(3) $$\overset{n}{\underset{k=2}{\sum }}\left|d_{k}^{p_{l}}\right|+\overset{n}{\underset{k=2}{\sum }}\left|d_{k}^{p_{r}}\right|$$

as an indicator of the overall JSN progression of KOA patients. This value was used to perform the clustering.

For data clustering, a centroid-based (k-means [20]), a connectivity-based (k-medoids [21]), and distribution-based clustering method (hierarchical clustering [22]) were employed, whereas the Davies Bouldin index [23] was used to evaluate the optimal number of clusters. Further investigation was performed in order to determine the optimal number of clusters that will be adopted. In our cases, 2 clusters were finally chosen, as described in Section 3.3.1. Data resampling was performed in order to cope with the significantly imbalanced classes. To this end, the size of the majority cluster was reduced to the number sample of the minority one.
**Algorithm 1** Pseudoalgorithm of the clustering processInput: JSM measurements of the first five visits Output: Labeled data1. **For each** patient *p* ∈ 𝒫: Calculate the differences between the consecutive JSM measurements: $d_{j}^{p}=m_{j}^{p}-m_{i}^{p},\;\forall i\in \left[1,\ldots ,n-1\right],\;j\in \left[2,\ldots ,n\right]$
Calculate the sum of the absolute differences: $\sum_{k=2}^{n}\left|d_{k}^{p}\right|$ **End for each** 2. **For each** clustering method m examined Perform clustering evaluation with Davies Boulding index and calculate the optimal number of clusters $C_{m}$. Perform clustering with $C_{m}$ clusters **End for each** 3. Return labels and evaluate the clustered data

#### 2.1.3. Feature Engineering

To develop a robust FS methodology, the combination of the outcomes of 6 FS techniques was employed to avoid bias. This methodology consists of two filter algorithms (Pearson correlation [24] and Chi-squared independence test [25]), one wrapper technique, which is based on recursive feature elimination (RFE) [26], and three embedded algorithms (random forest [27], light GBM (gradient boosting model) [28], and logistic regression with L2 penalty [29]). Specifically, a majority vote scheme shapes the basis of the feature ranking. Each of the six FS techniques was performed separately providing a list of selected features. A vote is assigned to each attribute each time it is selected by one of the FS techniques. The final feature ranking was decided with respect to the votes received. In addition, in case of equality the ranking was shaped from the feature importance of the performing FS technique (Algorithm 2). Let ${FSS}_{i},\;i=1,\ldots ,6$ be the subset of the ranked features of the i FS technique, M be the total number of features, and $V_{j},\;j=1,\ldots ,M$ be the total vote ranking for the j feature.
**Algorithm 2** Pseudoalgorithm of the feature selectionInput: Clinical data1. All features were normalized as described in the Pre-processing Section 2. **For each** feature j, Set $V_{j}=0$ **End for each** 3. **For each** FS technique i **For each** feature j If feature j is in $FSS_{i}$,
$$V_{j}=V_{j}+1$$**End if**                   **End for each** **End for each** 4. Rank features to descending order with respect to $V_{j}$. In case of equality the ranking is shaped from the feature importance of the best performing FS technique. **End**

#### 2.1.4. Data Classification

Six well-known classification algorithms were tested for the identification of the optimum model that achieves the highest accuracy on the test data:

- Gradient boosting model (GBM) is an ensemble ML algorithm, which can be used for classification or regression predictive tasks. Weak learners are used from GBM to produce strong learners through a gradual, additive, and sequential process. Hence, for the development of a new improved tree a modified version of the initial training data set is fitted in GBM [30,31];
- Logistic regression (LR) describes the relationship of data to a dichotomous dependent variable. LR is based on the logistic function (1). This model is designed to describe the data with a probability in the range of 0 and 1 [32]:

(4) $$f\left(x\right)=\frac{1}{1+e^{-x}},\;\mathrm{where}\;x\in \left(-\infty ,\;+\infty \right)\;\mathrm{and}\;0\leq f\left(x\right)\leq 1;$$

- Neural networks (NNs), both shallow and deep NNs were employed. NNs are based on a supervised training procedure to generate a nonlinear model for prediction. They consist of layers (e.g., input layer, hidden layers, and output layer). Following a layered feedforward structure, the information is transferred unidirectionally from the input layer to output layer through the hidden layers [33,34,35];
- Naïve Bayes Gaussian (NBG) employs the Bayes theorem. This probabilistic classifier presents strong independence assumptions between the variables/features given the class. Furthermore, this model embraces the assumption that the data follow the Gaussian distribution [36,37];
- Random forest (RF) belongs in the ensemble learning methods and is based on decision trees. This model constructs a large number of decision trees. Every decision tree denotes a class prediction. Thus, the class with the most votes represents the model’s prediction [38,39];
- Support vector machines (SVMs) are another supervised learning model [40,41]. SVMs target to create the hyperplane, which is a decision boundary between two classes that enables the prediction of labels from one or more feature vectors. The main aim of SVMs is to maximize the class margin that is actually the distance between the closest points (support vectors) of each class [42].

#### 2.1.5. Post-Hoc Interpretation/Explainability

In this study the SHapley Additive exPlanations (SHAP) were employed to rank features in terms of their impact on the final ML outputs. SHAP builds a mini explainer model for a single row-prediction pair that explains how this prediction was achieved. It is based on optimal shapley values from coalitional game theory that indicate how to fairly distribute the impact on model’s prediction among the features [43].

## 3. Evaluation

### 3.1. Medical Data

Data from the osteoarthritis initiative (OAI) database (available upon request at https://nda.nih.gov/oai/) were used in this study. Specifically, only clinical data from the baseline from all individuals without or being at high risk to develop KOA in at least one knee were included. In total, 725 features from 9 feature categories were considered as possible risk factors for the prediction of JSN as shown in Table 1. Clustering was performed on the JSN progression represented by the JSM measures (especially using the variables V00XRJSM, V01XRJSM, V03XRJSM, V05XRJSM, and V06XRJSM of the OAI from the first five visits) to group patients into two clusters (non-progressing patients and those whose JSN changes over time).

The available data from the baseline visit were divided into 9 categories (Table 1): (i) Anthropometrics, (ii) behavioral, (iii) symptoms, (iv) quality of life, (v) medical history, (vi) medical imaging outcomes, (vii) nutrition, (viii) physical exam, and (ix) physical activity. The first category contains anthropometric characteristics, such as body mass index, weight, and height. The behavior category concerns the habits and sociability of the participant. The symptoms category also contains all features that are associated with pain and any dysfunction. The quality-of-life category refers to variables that represent the participation of the individual to social events and activities. The medical history category includes features related to the medical history of the participants and of their family and whether they have received a medical prescription in specific time periods. Another category is the medical imaging outcomes which come after clinical evaluation with imaging such as X-rays. In addition, in the category of physical examination, we included all the characteristics related to the examination of a participant (such as hand and knee exam), various biomechanical measurements, and field tests. Finally, the category of physical activity includes all variables that relate to the individual activity, such as household activities and leisure activities.

### 3.2. Evaluation Methodology

The proposed methodology was applied in the context of predicting the JSN progression in patients with KOA by using the medical data derived from the dataset (Section 3.1). Initially, the methodology was applied for each leg separately and, consecutively, for both legs combined.

Following the clustering approach presented in Section 2, the JSN progression data were used as input to the clustering algorithm resulting in the identification of the patient groups with/without JSN progression. To this end, a comparative analysis of the various clustering methods employed in this stage was realized. The Davies Bouldin index was also used for an automatic identification of clusters within the dataset and to identify the magnitude of the variation in the JSM measures of patients. The parameter settings for the clustering methods are shown in Table 2.

The proportion of 70–30% was chosen for splitting the data set to training set and testing set, respectively, with normalization upon the features. The models evaluation was performed on the medical dataset presented in Section 3.1. Hyper parameter tuning was applied to most of the aforementioned models with grid search and 3-fold cross validation. Specifically, the involved hyper parameters are presented in Table 3 for each model. The prediction models were evaluated in subsets of features with increasing dimensionality.

### 3.3. Results and Discussion

#### 3.3.1. Clustering Results

**Figure 3:**
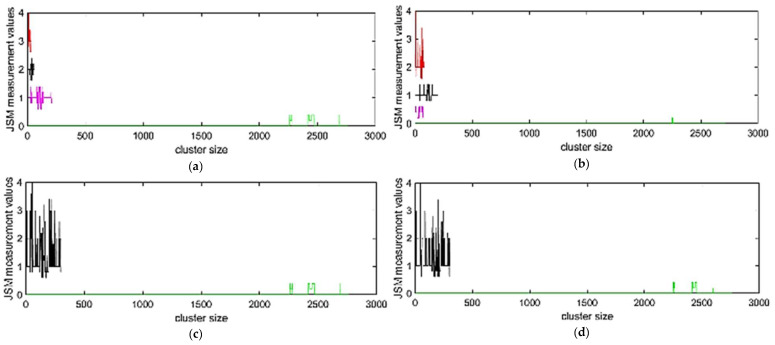

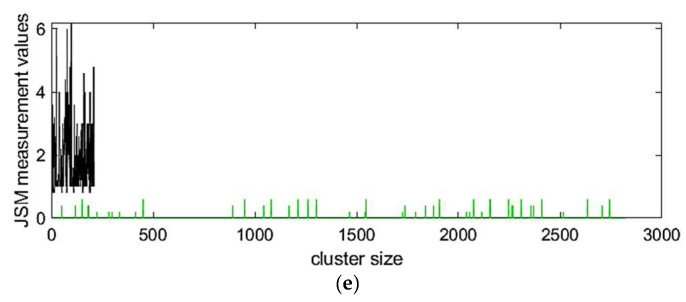
K-means clustering results of (**a**) left knee with Davies Bouldin index; (**b**) right knee with Davies Bouldin index; (**c**) left knee with 2 clusters; (**d**) right knee with 2 clusters; and (**e**) left and right knees combined with Davies Bouldin index.

In the clustering process, an identification of the clusters among the patients was attempted initially by using the Davies Bouldin index. Specifically, with this approach, four clusters (Table 4) were identified in most of the methods, grouping the patients to those with zero, low, medium, and high alterations in JSM measures in the case of the left leg or right leg, separately. However, the generated clusters presented an imbalanced allocation of the patients. Specifically, Cluster 1 proved to be significantly bigger compared to the other three identified clusters for both left and right legs (Figure 3a,b). For instance, for the right leg, it can be observed that a percentage of the patients with low JSN alterations were erroneously clustered in Cluster 1, which represents the patients with stable condition or without KOA. To overcome these problems, another clustering with only 2 clusters was performed. This issue was not observed during the clustering of both legs combined. In this case, 2 clusters were identified by using the Davies Bouldin index (Figure 3e). From the tested clustering approaches, k-means method was adopted based on the clustering results (Table 4 and Table 5). K-means achieved better clustering among patient groups. Regarding the identified clusters, the large one includes patients with stable JSN progression or patients that did not present KOA at all in their left and/or right leg, while the second one includes patients with alterations to JSN measures (Figure 3c–e).

#### 3.3.2. Feature Selection Results

**Figure 4:**
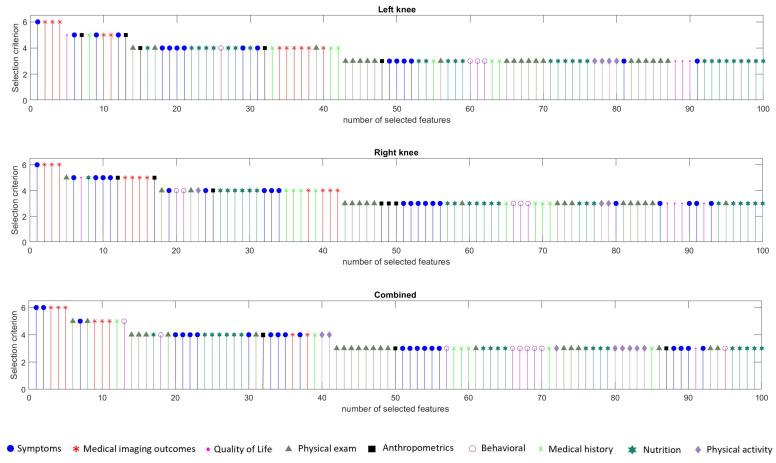
The first 100 features selected for the left (**top**), the right knee (**middle**), and both legs (**down**).

Figure 4 illustrates the first 100 features that are selected based on the proposed FS approach separately for the first strategy as well as for the second strategy. From the analysis of the results (Figure 4), we have concluded that the feature categories with the highest contribution seem to come from the symptoms’ category and the category of medical imaging outcomes. Indeed, in all cases there is a feature or two from the symptoms’ category that were selected first. Then, three imaging outcomes were selected on all three cases. In total, 21, 19, and 20 features of the first 40 selected in the left knee, right knee, and both knees combined, respectively, come from either the symptoms or the imaging outcomes category. Other contributing factors proved to be the nutrition and physical exam outcomes since approximately 20 out of the 100 features were selected in each case. Features from the anthropometrics and medical history categories were selected in all cases. Overall, the main outcome of this analysis is that a combination of heterogeneous features from almost all feature categories is necessary for an accurate prediction of JSN. This highlights that there is a need for a multi-parametric approach in order to handle the complexity and heterogeneity of the available data.

#### 3.3.3. Classification Results

**Figure 5:**
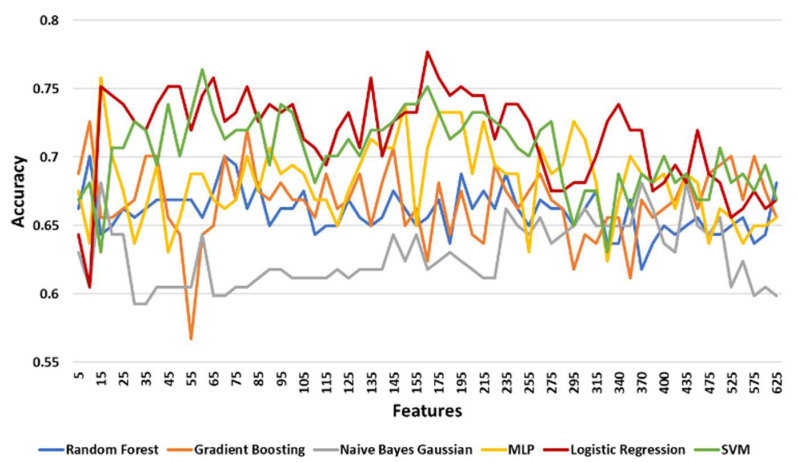
The accuracy of models over test set for increasing number of features for the left leg. Results are shown with a step size of 5 (two features added at each step).

**Figure 6:**
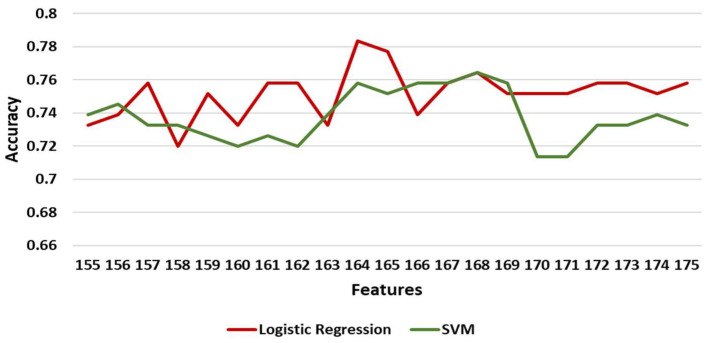
The accuracy of LR (logistic regression) and SVM at [155, 175] features over the test set for left leg. Results are shown with a step size of 1 (one feature added at each step).

Table 6 shows the maximum, minimum, and mean accuracy along with the standard deviation achieved by the models over the test set for increasing the number of features for the left leg. Figure 5 shows the alterations in the achieved accuracy over the test set with respect to the number of features (with a step of 2) for the left leg. For the left leg, the LR model performed better than the others with a maximum accuracy of around 77.7% for 165 features (Table 6, Figure 5). However, NNs and SVM had a comparative performance with a 75.8% and 76.4% maximum accuracy, respectively. To identify the exact number of features where the prediction accuracy is maximized, the two best performing models (LR and SVMs) were tested in the range of 155 and 175 features with a step of 1 with the results shown in Figure 6. The LR model performed best (≅78.3%) at 164 features. For this performance the following hyperparameters were used: Maximum number of iterations 100, intercept scale 1, L2 penalty, Newton-cg solver with reuse of the previous solution as initial one, and tolerance 0.0001.

**Figure 7:**
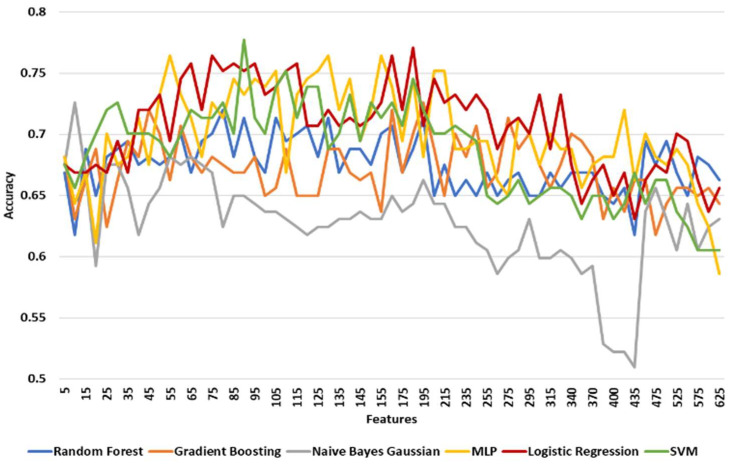
The accuracy of prediction models over test set for various number of features for the right leg. Results are shown with a step size of 5 (two features added at each step).

**Figure 8:**
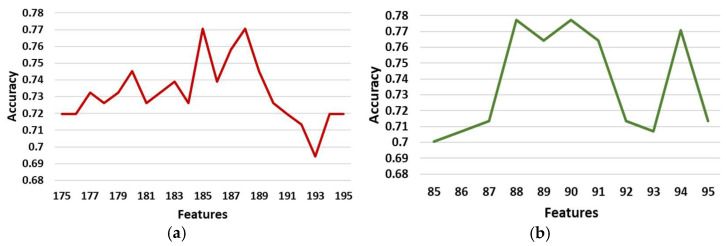
The performance evaluation of (**a**) LR in the range of 175–195 features and (**b**) SVM in the range of 85–95 features. Results are shown with a step size of 1 (one feature added at each step).

For the right leg a similar approach was adopted. Table 7 shows the maximum, minimum, and mean accuracy with the standard deviation achieved by the models over the test set for increasing the number of features for the right leg. Figure 7 shows the alterations in the achieved accuracy over the test set with respect to the number of features with a step of 2 for the right leg. The SVM model presented the best performance by achieving the maximum accuracy (≅77.7%) for 90 features (Table 7). However, the LR and NNs models accomplished an adequate performance (Figure 7). Specifically, the LR model achieved a higher mean accuracy (≅70.7% ±0.036) with a lower standard deviation compared to the results of the model (≅68.6%±0.039) (Table 7). To this end, these two models were re-evaluated for features in the neighborhoods, $U_{LR}\left(185,10\right)$ and $U_{SVM}\left(90,5\right)$ with a step of 1 feature at a time. LR achieved its best performance (≅77.1% accuracy) at 185 and 188 features while the SVM model reached its maximum accuracy of 77.7% with 88 and 90 features (Figure 8). The SVM model’s hyperparameters that achieved the best performance are the following: A linear kernel, regularization parameter at 0.1, tolerance at 0.001, and cache size at 200.

**Figure 9:**
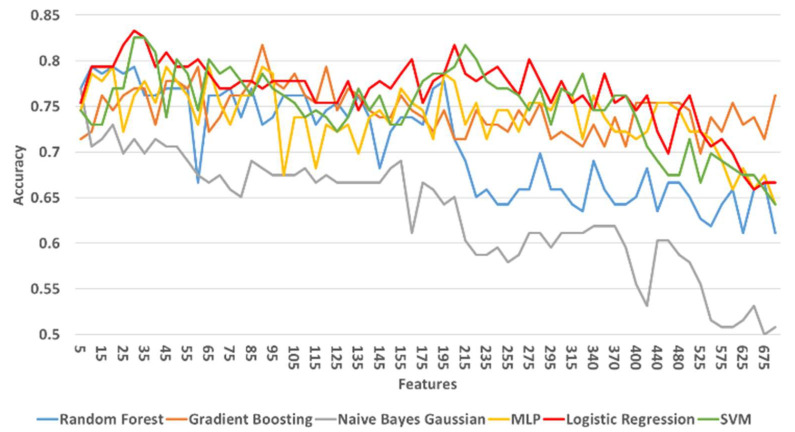
The accuracy of prediction models over test set for various number of features for the left and right legs combined. Results are shown with a step size of 5 (two features added at each step).

**Figure 10:**
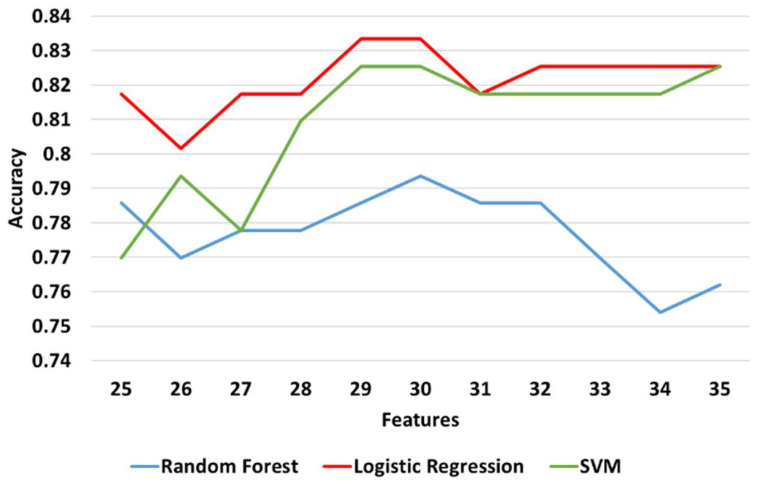
The accuracy of LR, RF (random forest), and SVM from 25 to 35 features over the test set for left and right legs combined. Results are shown with a step size of 1 (one feature added at each step).

Table 8 shows the maximum, minimum, and mean accuracy achieved among with the standard deviation by the models over the test set for a various number of features for both right and left legs combined. Figure 9 shows the alterations in the achieved accuracy over a test set for various number of features for both right and left legs combined. The results show that the LR model performed better compared to the other models by reaching the maximum accuracy (≅83.3%) for 30 features, as illustrated in Table 8 and Figure 9. Nevertheless, SVM and RF showed a comparative performance. The aforementioned three models are re-evaluated in order to find the number of features that maximizes the accuracy. Hence, the models are tested in the neighborhood where all three models achieved their best performance U(30,5). From a more detailed analysis, LR remained the predictive model with the best performance (≅83.3%) for 29 features (Figure 10).

**Figure 11:**
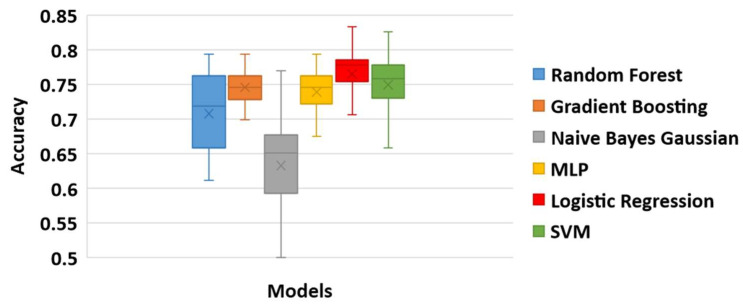
The box plot of the prediction models based on their performance for the right and left legs combined.

Overall, LR presented a stable performance (Figure 11, 77%±0.04) reacing the maximum accuracy at 29 features (83.3%). The hyperparameters of the LR model with the best performance were identical to the ones presented for the case of the first strategy. A generalized linear model such as LR has accomplished the best performance in our study, indicating that the power of the proposed methodology is not so much dependent on the complexity of the learning model but actually lies on the effective and robust mechanism of selecting important risk factors. Identifying robust predictive risk factors from a high dimensional feature space (such as the OAI dataset) is crucial since it enhances our understanding of KOA progression and therefore contributes to the development of robust prediction tools.

From the aforementioned classification results on the two proposed strategies (analysis on separate legs and combined) the following remarks can be drawn. Training predictive models using data from one of the two legs leads to inferior results compared to the performance of the model that is trained on data coming from both legs. This can be attributed to the fact that a predictive model trained on data only from the right leg ignores any JSN progression that might happen to the left leg. Due to complex interactions that occur in the dynamics of both legs, predictive models that are trained on data from a single leg are based on partial knowledge of the problem and thus lead to inferior results while requiring a larger number of features. The second strategy takes into account information from both legs and therefore leads to a well-defined data classification problem in which the non-progressors do not experience any JSN progression in any of their legs, whereas the progressors’ class incudes data from patients that experience JSN progression in at least one of their legs or both. This data problem proved to be more effectively handled by the proposed methodology with a 83.33% prediction accuracy at the first 29 features.

The need for applying data under-sampling on the dataset could be considered as a limitation of our study. Alternative data resampling algorithms (including more advanced data augmentation techniques such as generative adversarial networks) have been identified as a future research direction. The use of additional evaluation metrics (other than accuracy) such as precision, recall, or F score would also be beneficial for dealing with the observed data imbalance problem.

#### 3.3.4. Post-Hoc Explainability Results

**Figure 12:**
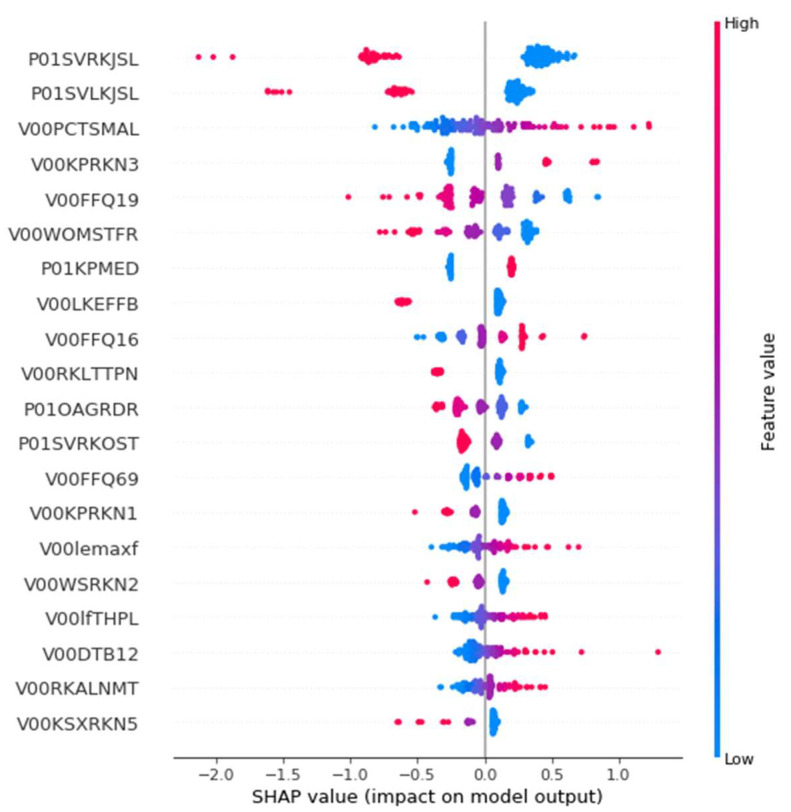
The distribution of the features’ impact on LR model output for the OAI (osteoarthritis initiative) dataset with 29 features across all instances.

**Figure 13:**
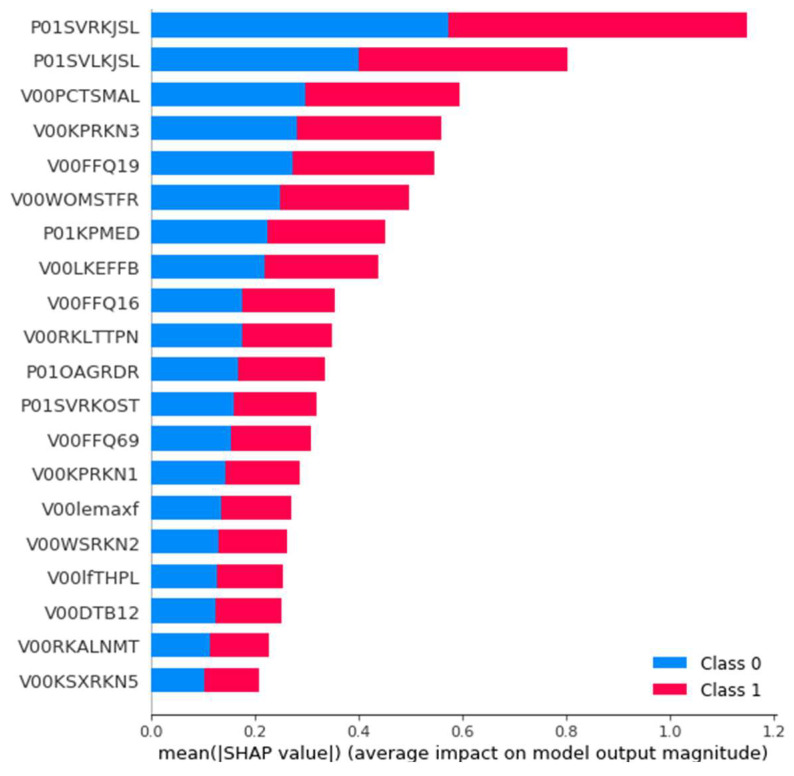
The average impact magnitude of 29 features on the LR model output for the OAI dataset for all instances.

To explain the impact of the selected features to the outcomes of the employed best prediction model, the SHAP method was used. SHAP was applied to the LR model which was trained on the selected 29 features that come from both legs. In Figure 12, the features are sorted by the sum of SHAP value magnitudes over all samples. The SHAP values are used to indicate the distribution of each feature’s impact on the model’s output. Specifically, the feature value is represented by color, with the red color corresponding to a high impact while the blue to a low impact. For instance, a high P01SVRKJSL value (evidence of knee lateral joint space narrowing) lowers the predicted status of the subjects. The features P01SVLKJSL, V00FFQ19, V00WOMSTFR, V00LFEFFB, V00RKLTTPN, P01OAGRDR, P01SVRKOST, V00KPRKN1, V00WSRKN2, and V00KSXRKN5 present a similar behavior. On the contrary, V00FFQ16 (how often the patient ate dishes with rice in the past 12 months) has a positive effect on the prediction outcome. Similar behavior was identified for the features V00PCTSMAL, V00KPRKN3, P01KPMED, V00FFQ69, V00lemaxf, V00lfTHPL, V00DTB12, and V00RKALNMT. Figure 13 illustrates the mean absolute value of SHAP values for each feature as a standard bar plot, which depicts the SHAP global feature importance. We observe that each feature has the same impact on both classes. Furthermore, the most important features that affected the prediction output were the P01SVRKJSL, P01SVLKJSL, and V00PCTSMAL.

## 4. Conclusions

The main objective of this study was the accurate prediction of JSN in KOA patients based on a machine learning pipeline trained on multimodal data from the OAI (725 features in total were considered). To identify and group patients with and without JSN progression a clustering process was initially performed on the JSN progression based on the JSM outcomes of patients over the first five visits. Afterwards, for the identification of the most important features for the related clusters discrimination (progressing versus non-progressing patients), a hybrid feature selection technique was employed. Finally, the selected features were employed for the training of various ML models in order to predict JSN in KOA patients. The outcome of the ML models indicated that the LR model achieved the best performance for the left leg with a 78.3% accuracy for 164 features, while for the right leg, the SVM model dominated with a 77.7% accuracy for 88 and 90 features.

However, the best overall performance was achieved by the second strategy where the data from both legs were combined. Specifically, the LR model achieved a 83.3% accuracy for a significantly lower number of features (29). This study was not only focused on the development of prediction models, but also aimed to reveal significant insights regarding the nature of the predictive risk factors that were identified as important. Through this analysis, we concluded that a blend of heterogeneous features from almost all feature categories is necessary in order to maximize the performance and prediction accuracy of the models. The nature of the selected features along with their impact on the prediction outcome (via SHAP) were also discussed to increase our understanding of their effect on JSN progression. Future work should focus on incorporating morphological knee features as an additional feature category that could potentially increase the performance of the predictive models. These features can be extracted by employing deep learning algorithms for image processing. Alternative data clustering algorithms, such as self-organizing maps (SOM) could also be explored to improve the clustering performance of the proposed methodology, leading to more informative and distinct data classes.

## Figures and Tables

**Table 1:** Maim categories of the feature subsets considered in the proposed methodology.

Category	Description	Number of Features
Anthropometrics	Includes measurements of participants such as height, weight, BMI (body mass index), etc.	37
Behavioral	Questionnaire results which describe the participants’ social behaviour	61
Symptoms	Includes variables of participants’ arthritis symptoms and general arthritis or health-related function and disability	108
Quality of life	Variables which describe the quality level of daily routine	12
Medical history	Questionnaire results regarding a participant’s arthritis-related and general health histories and medications	123
Medical imaging outcome	Variables which contain medical imaging outcomes (e.g., osteophytes and joint space narrowing (JSN))	21
Nutrition	Variables resultfrom the use of the modified Block Food Frequency questionnaire	224
Physical exam	Variables of participants’ measurements, performance measures, and knee and hand exams	115
Physical activity	Questionnaire data results regarding household activities, leisure activities, etc.	24
	Total number of features:	725

**Table 2:** Parameter settings for clustering methods.

Clustering Method	Parameters
K-Means	City block distance, 5 replicates
K-Medoids	City block distance, 5 replicates
Hierarchical	Agglomerative cluster tree, Chebychev distance, farthest distance between clusters, 3 maximum number of clusters

**Table 3:** Hyper parameter settings for tuning. GBM: Gradient Boosting Model; LR: Logistic Regression; NN: Neural Networks; NBG: Naïve Bayes Gaussian; RF: Random Forest; SVM: Support Vector Machine.

Classification Model	Hyper Parameters Tuning
GBM	The number of boosting stages to perform from 10 to 500 with 10 step size The maximum depth of the individual regression estimators from 1 to 10 with 1 step size The minimum number of samples required to split an internal node: 2, 5 and 10 The minimum number of samples required to be at a leaf node: 1, 2 and 4 The number of features to consider when looking for the best split: $\sqrt{n_{features}}$ or $\log_{2}\left(n_{features}\right)$
LR	The inverse of regularization strength was tested on 0.001, 0.01, 0.1, 1, 2, 3, 4, 5, 6, 7, 8, 9, 10 Algorithm to use in the optimization problem was set to 4 different solvers that handle L2 or no penalty, such as ‘newton-cg’, ‘lbfgs’, ‘sag’ and ‘saga’ A binary problem is fit for each label or the loss minimized is the multinomial loss fit across the entire probability distribution, even when the data is binary With and without reusing the solution of the previous call to fit as initialization
NN	Both shallow and deep structures were investigated Hidden layers varying from 1 to 3 with different number of nodes per layer (50, 100, 200) Activator function: Relu and tanh Solver for weight optimization: adam, stochastic gradient descent, stochastic gradient-based optimizer proposed by Kingma, Diederik, and Jimmy Ba and an optimizer in the family of quasi-Newton methods L2 penalty (regularization term) parameter: 0.0001 and 0.05 The learning rate schedule for weight updates was set as a constant learning rate given by the given number and as adaptive by keeping the learning rate constant to the given number as long as training loss keeps decreasing.
NBG	-
RF	The number of trees in the forest from 10 to 500 with 10 step size The maximum depth of the tree from 1 to 10 with 1 step size The minimum number of samples required to split an internal node: 2, 5 and 10 The minimum number of samples required to be at a leaf node: 1, 2 and 4 The number of features to consider when looking for the best split: $\sqrt{n_{features}}$ or $\log_{2}\left(n_{features}\right)$ With and without bootstrap
SVM	The regularization parameter was tested on 0.001, 0.01, 0.1, 1, 2, 3, 4, 5, 6, 7, 8, 9, 10 Kernel type was set to linear, polynomial, sigmoid and radial basis functions

**Table 4:** Clustering results of each case with the Davies Bouldin index. The best results are indicated with bold.

Clustering Method	Number of Clusters			Cluster Elements		
	*Left*	*Right*	*Both*	*Left*	*Right*	*Both*
K-Means	4	4	2	[2763,209,62,28]	[2733,199,84,50]	**[2822,209]**
K-Medoids	4	4	2	[2763,209,62,28]	[2733,199,84,50]	[2822,209]
Hierarchical	4	3	2	[2960,74,24,4]	[2989,68,9]	[3016,15]

**Table 5:** Clusters adopted in our study for each case. The best results are indicated with bold.

Clustering Method	Number of Clusters			Cluster Elements		
	*Left*	*Right*	*Both*	*Left*	*Right*	*Both*
K-Means	2	2	2	**[2763,299]**	**[2764,302]**	**[2822,209]**
K-Medoids	2	2	2	[2763,299]	[2764,302]	[2822,209]
Hierarchical	2	2	2	[3034,28]	[2989,77]	[3016,15]

**Table 6:** Maximum, minimum, and mean accuracy of prediction models over the tested set for the left leg. The best results are indicated with bold.

Prediction Model	Maximum Accuracy	Minimum Accuracy	Mean Accuracy	Standard Deviation
Gradient Boosting	0.72611	0.56688	0.66707	0.02622
Logistic Regression	**0.77707**	0.60510	**0.71540**	0.03353
NNs (Neural Networks)	0.75796	0.62420	0.68234	0.02933
Naïve Bayes Gaussian	0.68153	0.59236	0.62794	0.02301
Random Forest	0.70064	0.61783	0.65989	0.01616
SVM	0.76433	0.63057	0.70377	0.02783

**Table 7:** Maximum, minimum, and mean accuracy of prediction models over the tested set for the right leg. The best results are indicated with bold.

Prediction Model	Maximum Accuracy	Minimum Accuracy	Mean Accuracy	Standard Deviation
Gradient Boosting	0.72611	0.61783	0.67172	0.02445
Logistic Regression	0.77070	0.63057	**0.70691**	0.03560
NNs	0.76433	0.58599	0.69983	0.03858
Naïve Bayes Gaussian	0.72611	0.50955	0.62774	0.03926
Random Forest	0.71975	0.61783	0.67577	0.02217
SVM	**0.77707**	0.60510	0.68598	0.03929

**Table 8:** Maximum, minimum, and mean accuracy of prediction models over the tested set for the left and right legs combined. The best results are indicated with bold.

Prediction Model	Maximum Accuracy	Minimum Accuracy	Mean Accuracy	Standard Deviation
Gradient Boosting	0.81746	0.69841	0.74591	0.02449
Logistic Regression	**0.83333**	0.65873	**0.76503**	0.03725
NNs	0.79365	0.64286	0.73870	0.03470
Naïve Bayes Gaussian	0.76984	0.50000	0.63300	0.06331
Random Forest	0.79365	0.61111	0.70755	0.05645
SVM	0.82540	0.64286	0.74928	0.04223

## Data Availability

Data from the osteoarthritis initiative (OAI) database (available upon request at https://nda.nih.gov/oai/).
